# Place Still Matters: Social Vulnerabilities, Place-Level Disadvantage, and Food Insecurity during COVID-19

**DOI:** 10.3390/nu15061430

**Published:** 2023-03-16

**Authors:** Kevin M. Fitzpatrick, Casey T. Harris, Don Willis, Amber Obermaier, Grant Drawve

**Affiliations:** 1 Department of Sociology and Criminology, University of Arkansas, Fayetteville, AR 72701, USA; 2 College of Medicine, University of Arkansas for Medical Sciences Northwest, 1125 N. College Ave., Fayetteville, AR 72703, USA

**Keywords:** food insecurity, COVID-19, health and community

## Abstract

COVID-19 variants continue to create public health danger impacting mortality and morbidity across the United States. The spillover effects of COVID-19 on the economy and social institutions pose a significant threat to broader wellbeing, including the food security of millions across the country. We aim to explore whether the context of place matters above and beyond individual and social vulnerabilities for food insecurity. To do so, we employ a multi-level framework using data from a survey of over 10,000 U.S. adults from March 2020 with American Community Survey (ACS) and John Hopkins COVID Dashboard county-level data. We find nearly two in five respondents were food insecure by March of 2020 with disparities across race, nativity, the presence of children in the home, unemployment, and age. Furthermore, we note that individuals living in more disadvantaged communities were more likely to report food insecurity above and beyond individual and social vulnerabilities. Overall, food insecurity is driven by complex, multi-level dynamics that remain a pressing public health concern for the current—but also future—public health crisis.

## 1. Introduction

The first case of COVID-19—the disease caused by the novel coronavirus (SARS-CoV-2)—was confirmed in the United States (U.S.) by mid-January 2020. The first COVID-19 death was recorded less than a month later. After nearly three years, over 1.1 million U.S. deaths have been attributed to this deadly virus [1]. Although the virus and its variants continue to create public health danger impacting U.S. mortality and morbidity, the spillover effects of COVID-19 on the economy and social institutions also pose a significant threat to broader wellbeing, including the food security of millions across the country.

COVID-19 disrupted America’s economy, social life, and food systems. Recent reports have noted that food insecurity decreased for certain groups—namely households with children—in 2021 after a host of relief measures were enacted by policymakers, including Economic Impact Payments, expanded Child Tax Credits, improved unemployment insurance, and expanded food assistance [2,3]. Despite such improvements among families with children [3,4,5,6,7,8], legislators have allowed many of the measures to expire, potentially undoing much of the progress these policies had made in 2021. Meanwhile, early in the pandemic, prior to the enactment and improvement of those relief measures, large numbers of persons became newly food insecure [4]. Thus, access to food amidst the ongoing COVID-19 pandemic remains a concern among policymakers, the public, and food chain suppliers as food scarcity has waxed and waned.

Food insecurity—the absence of “access by all people at all times to enough food for an active, healthy life” [9] (p. 2)—has been linked to a myriad of poor health outcomes for children, adolescents, and adults, including chronic disease, diabetes, iron deficiency, depression, anxiety, and poor oral health, making it a leading public health concern [10]. Most efforts to understand disproportionate experiences with food insecurity focus on individual and social vulnerabilities (e.g., personal resources, family type, employment) [7,10,11,12]. A smaller body of the literature has begun to highlight the role of place-level or contextual factors related to food insecurity [13,14,15,16,17]. Place is a basic component of lived experience and, as such, it is often taken for granted. Yet, place remains an important factor affecting how individuals secure food. In turn, the COVID-19 pandemic may have reorganized experiences of space and place: as infections and deaths rose, the public health measures for social distancing and quarantine may have restricted the way people moved through communities in ways that increased food insecurity risk for some people more than others. Contributing to the limited body of research focused on place and food insecurity we focus here on the role of context or place above and beyond the known individual-level risk factors for food insecurity during the early COVID-19 pandemic. Unlike individual measures, place-level variables capture context—the backdrop against which individual and social vulnerabilities exist. While individual and social vulnerabilities provide information about individual positionality, place-based measures provide information about the context within which individuals are positioned in their geographic spaces that might also impact food insecurity during times of public crisis.

Broadly, material hardships have not been uniform across time and space during the ongoing public health crisis. A growing body of empirical research shows that some groups have experienced significant burden while others have not, just as some regions, states, and cities have experienced wide disparities in food shortage and food insecurity while others have not [4,18,19,20]. For example, early in the pandemic, racial and ethnic minorities, persons experiencing unemployment, those who are younger, and families with children reported significant upticks in food and housing insecurity [8,11,18]. Many households that were already struggling to meet basic needs prior to COVID-19 encountered additional barriers that exacerbated or pushed them into food insecurity [21,22,23,24]. In turn, long lines at the food pantry, empty shelves at the grocery store, and limited access to social resource networks quickly became overwhelmed and underfunded amidst pandemic-related economic fallout [19,20].

Although individual and social vulnerabilities have been linked wide-ranging differences in food access, the question remains: which characteristics of locales (place) help explain individual food insecurity? For example, early research concerning the impact of COVID-19 on food insecurity levels across the United States clearly reveals regional disparities [4,25,26]. However, there remains considerable heterogeneity within regions, just as little is known about how place-level variables matter (e.g., contexts of poverty, pandemic conditions). Such gaps in knowledge raise the possibility that differential exposure across places to the virus’s impact throughout the pandemic may have created varying degrees of food insecurity for those living within them. Thus, while geographic patterns in food insecurity are not well understood, they remain an important part of the larger solution to systematically and rationally providing solutions that mitigate public health and natural disaster crises that can cripple food systems both locally and nationally.

In an effort to assess several important relationships between food insecurity risk factors, the current paper examines both individual and social vulnerabilities, the geographic distribution of COVID-19 cases, and place-level structural disadvantages within a multi-level framework. More broadly, we aim to explore whether or not characteristics of communities/places matter above and beyond individual and social food insecurity risk as it is adjudicated within the food insecurity literature. In doing so, we make two important advances beyond the prior literature. First, the current study simultaneously examines individual- and place-level predictors of food insecurity, rather than treating patterns of food insecurity as determined separately at micro- and macro-levels. Second, we advance research on pandemic-related social consequences by examining how food insecurity was associated with localized proximity to COVID-19 cases, in particular. As described below, the data employed here offer an important point of measurement during the early stages of the United States’ experience with the ongoing pandemic.

## 2. Materials and Methods

### 2.1. Sources of Data

We drew on three data sources for the current study. First, we used data from a large-scale survey of United States adults from March 2020 that documented food insecurity for over 10,000 adults. Important for our purposes, individual respondents were identified geographically through the online (Qualtrics Inc., Seattle, WA, USA), institutionally approved survey early in the pandemic (23 March 2020). The final sample of 10,368 adults was post-stratification-weighted by gender, age, race, income, and geography (state) to ensure the equitable contribution of respondents across their demographic and geographic strata relative to their representation in the overall population of the United States see [4]. We noted that these data reflect an early—but strategic—stage of the COVID-19 pandemic when there was still considerable diversity in exposure to the epidemic and related social problems (i.e., before saturation after multiple waves of infection). This allowed for a useful examination of the relationship between disparities in individual-level and place-level factors that may have created greater homogeneity in food insecurity. Likewise, examining such an early stage provided insight into those factors most associated with acute “shocks” to food systems during public health crises by identifying the most vulnerable individuals and the types of communities in which they live.

Second, we captured information about the communities in which respondents live using the United States Census Bureau’s five-year (2014–2018) American Community Survey summary files. Third, we drew on the John Hopkins University COVID-19 Dashboard that identifies the number of confirmed cases and deaths by geography (county) in the United States each day [27]. This data source has served as a primary indicator of the coronavirus’s spread since early in the pandemic and provides estimates of both cases and deaths at multiple geographic levels (e.g., states, counties).

All data were paired using federal information processing standards (FIPS) codes for each state and county. Survey respondents reported their residential zip code, which were then used with the U.S. Department of Housing and Urban Development crosswalk file to assign counties [28]. To reduce instability in our estimates, we restricted our sample to only include those counties in which there are 10 or more respondents. The final sample included 6825 United States adults nested within 199 counties.

### 2.2. Dependent Variable

Our dependent variable was food insecurity, measured using the standard, ten-item USDA Adult Food Security Module [9] which asks the following: “Thinking about your experience with food over the last 3 months”. While this question differs from the standard last twelve months or last thirty days assessment, a three-month window was deemed appropriate for capturing food insecurity at the onset of the pandemic (survey administration was late-March 2020). Respondents were subsequently provided a series of ten items to which they could respond with “often true”, “sometimes true”, or “never true”, including (1) I worried whether my food would run out before I got money to buy more; (2) The food that I bought just didn’t last and I didn’t have money to get more; (3) I couldn’t afford to feed myself or family a balanced meal because I couldn’t afford it; (4) I relied on only a few kinds of lost-cost food to feed myself or family because I was running out of money to buy food. The remaining questions could be responded to with “yes”, “no”, including the following: (5) cut the size of your meals because there wasn’t enough money for food; (6) eaten less than you felt you should because there wasn’t enough money for food; (7) skipped meals because there wasn’t enough money for food; (8) been hungry but didn’t eat because there wasn’t enough money for food; (9) lost weight because there wasn’t enough money for food, and (10) did not eat for a whole day because there wasn’t enough money for food. All affirmative responses were coded as 1 with non-affirmative responses coded as zero. Consistent with the previous use of this measure, respondents who answered in the affirmative to three or more items were considered food insecure (coded as 1), while those who answered in the affirmative to two or fewer items were considered food secure (coded as zero).

### 2.3. Independent Variables

We included the following individual and social characteristics that have been shown to impact food insecurity, including a dummy variable for female and whether a respondent was non-Hispanic Black, Hispanic, non-Hispanic Asian, or non-Hispanic Other race (non-Hispanic White serves as the reference). Additionally, we included dummy variables for whether respondents were foreign born, married, and/or had children present in their house at the time of response. Each respondent’s age (in years) and whether a respondent was unemployed at the time of the survey were also measured. A full list of the independent and dependent variables is shown in Table 1 below.

Central to the current study, individuals were nested within their residential counties, and we controlled two important characteristics of those places (place-level variables) that might impact food insecurity early in the pandemic. First, we included a measure of each county’s poverty rate for the population between the ages of 18 and 64 (% in poverty). Second, to account for food insecurity as a function of early COVID-19 case proximity, we included a measure of the confirmed neighboring county cases per 100,000 that captured the presence of coronavirus in each respondent’s county of residence and all surrounding counties. Queen’s 1st order contiguity was used to identify immediate neighboring counties that share a common edge or a common vertex. These were drawn from the John Hopkins University dashboard [27]. All geographic coding was done in ArcGIS™ Pro 2.6 and GeoDa [29]. Again, we noted that the temporal period for the current study reflects an early point in the pandemic when saturation of coronavirus cases had not yet occurred and there was considerable variation in the relative proximity of cases across geographic space.

### 2.4. Data Analysis

We began the analysis by describing the overall presence of food insecurity among survey respondents in March 2020, as well as the characteristics of individuals across each of our independent variables. The purpose here was to describe the overall prevalence of food insecurity and other individual and social vulnerabilities, as well as place-level variables that captured features of the counties in which surveyed individuals lived in March 2020. Second, we constructed a series of mixed-effects (multi-level) logistic regression models predicting food insecurity as a function of individual and social vulnerabilities alongside place-level variables. Because respondents living within the same spaces share underlying similarities, residual errors were likely to be correlated within counties in our sample, violating the assumption of independence necessary for ordinary least squares models and producing mis-specified standard errors. Instead, we employed mixed-effects models that accounted for the nesting of respondents within counties, adjusted the degrees of freedom to correctly represent the number of counties in our analysis used for conducting statistical tests, and allowed us to estimate the unique and independent relationship between both individual- and county-level (place) characteristics with the individual-level food insecurity outcome [30]. All covariates were grand-mean centered in order to provide a meaningful interpretation of the model constant [31] and were estimated in Stata 15 using the mixed command. The purpose of this second stage of the analysis was to examine whether those place-level (county) characteristics mattered in addition to individual-level food insecurity risk factors.

## 3. Results

In Table 1, we present the survey-weighted means, proportions, and standard errors for all of our independent and dependent variables. We found that food insecurity was exceedingly high during the mid-to-late March 2020 period with 39 percent of respondents reporting some degree of food insecurity. Roughly half of respondents were female, and most were non-Hispanic White (54 percent) or Hispanic (21 percent). Individuals that were foreign born represent only a small fraction of respondents (13 percent), and just less than half of the sample reported being married at the time of interview (44 percent). Roughly a quarter of respondents indicated that a child was present in their home (26 percent) and about one-in-five indicated reported being unemployed (20 percent). The average age was just over 46 years. Additionally, we found that the poverty rate in respondents’ counties was 12.4 percent, which was slightly higher than the national average of 11.8 percent for the same years [32]. Respondents, on average, lived in counties with about 370 confirmed COVID-19 cases per 100,000 people in the surrounding counties.

In Table 2, we present results from the multi-level logistic regression models. We present both the coefficients and odds ratios for individual food insecurity as a function of individual-level and place-level (county) vulnerabilities. We found that, compared to White respondents, Black (b = 0.363, OR = 1.437) and Hispanic (b = 0.264, OR = 1.302) individuals were more likely to report food insecurity, while Asian respondents were less likely to do so (b = −0.372, OR = 0.689). Foreign-born individuals (b = 0.328, OR = 1.389), persons who had children present in the home (b = 0.521, OR = 1.683), and persons who were unemployed (b = 0.593, OR = 1.810) were also more likely to indicate being food insecure in the first three months of 2020. Older respondents were less likely to report food insecurity compared to their younger counterparts (b = −0.036, OR = 0.964).

As a central contribution of the current study, Table 2 also reveals that respondents living in counties with higher poverty rates were more likely to indicate they were personally food insecure, as well (b = 0.029, OR = 1.029). Thus, for every single point increase in the percentage of the population in poverty at the county-level, the odds of a person reporting food insecurity increased by 2.9 percent. In contrast, the overall geographic proximity to COVID-19 cases was not associated with individual food insecurity.

## 4. Discussion

The COVID-19 pandemic has been compounded by overburdened food pantries, threats of eviction, and a limited welfare system that was already under strain. We found that nearly two in five respondents were food insecure by March of 2020, an early point in the pandemic in the United States. Our finding confirmed prior research indicating a sharp rise in food insecurity during the early period of the pandemic [4,8,11,26]. We also found support for the growing body of research showing that disparities in food insecurity early on in the pandemic largely mirror those that existed prior to it, including disparities by race, nativity, the presence of children in the home, unemployment, and age [4,7,8,26]. Policies that are focused on addressing food insecurity among vulnerable populations continue to be critical.

Although findings related to the prevalence and disparate experiences of food insecurity across individual and social vulnerabilities are important, this study was primarily motivated by the following question: which characteristics of locales (place) help explain individual food insecurity? However, place is a complex and multidimensional concept. While our study did not examine an exhaustive list of contextual factors, we focused instead on two critical pieces of that place in the context of a global pandemic: county level poverty and geographically proximate COVID-19 case rates. We found that the context of county-level poverty was significantly associated with food insecurity during the pandemic. This is consistent with research prior to the pandemic which found food insecurity to be associated with poverty at the neighborhood level [33], but somewhat divergent from other studies which found no association with concentrated disadvantage as an indicator of neighborhood socioeconomic status [17]. Critically, this study contributes the novel finding that county-level COVID-19 cases were not a significant predictor of food insecurity early in the pandemic, as well.

Although studies have noted the impact of the pandemic on food insecurity, and especially the policies that have accompanied it, no other studies to our knowledge have examined the association with COVID-19 cases as a predictor of food insecurity. These findings are somewhat surprising, suggesting that food systems were not disproportionately burdened in places with more confirmed early COVID-19 cases (i.e., those places bearing the early “shock” of COVID-19). However, these results are not sufficient to conclude that the severity of the pandemic is more broadly unrelated to food insecurity given that cases are only one indicator of severity at the county-level (especially early in 2020). Other indicators, such as hospitalizations or deaths might be better correlates of food insecurity given that they could be more closely connected with the economic circumstances of a place. Future research should consider the impact of COVID-related contextual factors for specific populations, such as low-income households, and examine whether food insecurity rates were heightened among already vulnerable groups.

However, our finding that COVID-19 cases is unrelated to food insecurity marks a contrast to other studies demonstrating that early proximity to the virus increases individual-levels of fear [34]. As such, the processes generating food insecurity—whether at the micro- or macro-level—would appear to be distinct from other social phenomena observed early in the pandemic. This may reflect qualitative differences in the accumulation of factors that produce individual disparities in access to food versus other social ills, or it could indicate there are a different set of temporal processes (i.e., lagged effects) that shape things such as food insecurity differently than fear relative to proximity to public health crises.

The present study has limitations that warrant further inquiry in future research. For one, we relied upon cross-sectional data that cannot establish causality. Moreover, our cross-sectional data limits our ability to assess relationships with food insecurity during an evolving and ever-changing global crisis, especially given the measures (e.g., direct payments) taken to address economic hardships later in the pandemic. Although we found that COVID-19 cases at the county-level were not associated with individual-level food insecurity, the pathways through which COVID-19 cases might shape food insecurity should be examined further. For example, higher case rates might trigger policy decisions shaping the economic circumstances of any given place, but perhaps those policies occur at a contextual level (e.g., state) left unexamined in this analysis.

Furthermore, higher cases might have a lagged effect on food insecurity. As the pandemic progressed, payments were made to families to alleviate the economic hardships. However, for places that had higher case rates, there may be long-term material hardships due to excess death, loss of social support/family members, closing of businesses, and medical debt, all of which were unobserved in this study. Understanding the potential long-term impact of living in a place where COVID-19 cases spiked and remained consistently high will require further research. Finally, while we add to a limited body of research examining contextual correlates of food insecurity, we acknowledge the work of researchers who emphasize that congruence also matters. That is, place matters, but the characteristics of place may matter differently for different individuals, households, or families [15]. While our own supplemental analyses (not shown, available upon request) found no statistically significant cross-level interactions or random slopes in our multi-level models, we recommend that as researchers continue to investigate the role that place plays in shaping food insecurity during the pandemic that considers the importance of congruence.

## 5. Conclusions

Despite these limitations this study provides support for three broad conclusions. First, food insecurity was much higher during the early period of the pandemic than it had been in the year prior. Second, early pandemic experiences of food insecurity were disproportionately felt among individuals who are Black or Hispanic, foreign-born, had children living in the household, were unemployed, or younger. Finally, individuals living in places with higher poverty rates were more likely to experience food insecurity, while persons living in places with more COVID-19 cases were not. Food insecurity is disproportionately experienced among many of the same groups who experience worse COVID-19 outcomes, including places with higher rates of poverty [35].

In sum, food insecurity is driven by complex, multi-level factors that remain a pressing public health concern that can better inform responses to similar public health crises. Yet, the pandemic did spark policy actions that have been demonstrated to have reduced the burden of food insecurity. For example, the expanded Child Tax Credit and unemployment insurance have both been associated with decreased food insecurity [36,37]. Yet, political decisions were made to allow these policy actions to expire [38,39]. In short, despite the complexity of food insecurity, it is not an inevitable or natural outcome. There are policies known to reduce it, and political actors have chosen to undo those policies rather than maintain them. Our study did not examine the impact of policies; however, our findings do support the idea that contexts of poverty matter for food insecurity. Policies aimed at poverty-reduction for communities—beyond individuals and households—may also be important for reducing food insecurity.

The ongoing COVID-19 pandemic presents challenges beyond the immediate risk of infection, hospitalization, and death. Our findings demonstrate that the consequences on social life extend to some individuals and communities more than others. Thus, there is no “one-size-fits-all” approach to solving food insecurity and other social ills as the determinants operate across multiple levels of our social fabric. Nevertheless, we remain hopeful that additional scholarship and, in turn, practical applications of important findings from this empirical work will guide policymakers and practitioners to establish both immediate solutions to food insecurity, as well as prepare for future challenges presented by similar public health crises.

## Figures and Tables

**Table 1:** Descriptive Statistics for Model Variables.

	Mean	Proportion	Std. Error
Dependent Variables (*n* = 6825):			
Food insecure (dummy)	-	0.39	0.01
Individual and Social Vulnerabilities (*n* = 6825):			
Female	-	0.50	0.02
White	-	0.54	0.02
Black	-	0.13	0.01
Hispanic	-	0.21	0.02
Asian	-	0.07	0.01
Other race	-	0.03	0.01
Foreign born	-	0.13	0.01
Married	-	0.44	0.01
Children present	-	0.26	0.01
Age	46.32	-	0.68
Unemployed	-	0.20	0.01
Place-Level Variables (*n* = 199 counties):			
% Poverty (18–64)	12.42	-	1.59
COVID-19 Neighbor Case Rate	370.05	-	26.82

Note: All means, proportions, and standard errors reported after employing svy: mean to adjust for post-stratification weights in Stata 15. To avoid skewed values, all place-level and spatial descriptive statistics are estimated from an aggregated database that includes only one of each county.

**Table 2:** Multi-Level Logistic Regression Models Examining Individual Food Insecurity as a Function of Individual, Social, and Place-Level Vulnerabilities.

	b	SE	Odds Ratio
Individual and Social Vulnerabilities:			
Female	−0.108	0.058	0.898
Black	0.363 ***	0.102	1.437
Hispanic	0.264 **	0.099	1.302
Asian	−0.372 **	0.137	0.689
Other race	−0.215	0.263	0.807
Foreign born	0.328 **	0.105	1.389
Married	−0.349 ***	0.064	0.705
Children present	0.521 ***	0.074	1.683
Age	−0.036 ***	0.002	0.964
Unemployed	0.593 ***	0.077	1.810
Place-Level Variables:			
Neighbor COVID-19 case rate	0.000	0.000	1.000
% Poverty (18–64)	0.029 ***	0.008	1.029
Constant	−1.035 ***	0.034	0.355
*n* (individual-level)	6825		
*n* (county-level)	199		

** *p* < 0.05; *** *p* < 0.01.

## Data Availability

Data, as part of the funded research, will be archived through Community and Family Institute Website cfi.uark.edu (accessed on 8 January 2023).
